# Transient Conductive Hearing Loss Regulates Cross-Modal VGLUT Expression in the Cochlear Nucleus of C57BL/6 Mice

**DOI:** 10.3390/brainsci10050260

**Published:** 2020-04-29

**Authors:** Takaomi Kurioka, Sachiyo Mogi, Taku Yamashita

**Affiliations:** Department of Otorhinolaryngology, Head and Neck Surgery, Kitasato University Sagamihara, Kanagawa 252-0374, Japan; mogi@med.kitasato-u.ac.jp (S.M.); tyamahns@kitasato-u.ac.jp (T.Y.)

**Keywords:** Auditory deprivation, Neuroplasticity, Cochlear nucleus, VGLUT, Synapse

## Abstract

Auditory nerve fibers synapse onto the cochlear nucleus (CN) and are labeled using the vesicular glutamate transporter-1 (VGLUT-1), whereas non-auditory inputs are labeled using the VGLUT-2. However, the underlying regulatory mechanism of VGLUT expression in the CN remains unknown. We examined whether a sound level decrease, without primary neural damage, induces cellular and VGLUT expression change in the CN, and examined the potential for neural plasticity of the CN using unilateral conductive hearing loss models. We inserted earplugs in 8-week-old mice unilaterally for 4 weeks and subsequently removed them for another 4 weeks. Although the threshold of an auditory brainstem response significantly increased across all tested frequencies following earplug insertion, it completely recovered after earplug removal. Auditory deprivation had no significant impact on spiral ganglion and ventral CN (VCN) neurons’ survival. Conversely, although the cell size and VGLUT-1 expression in the VCN significantly decreased after earplug insertion, VGLUT-2 expression in the granule cell lamina significantly increased. These cell sizes decreased and the alterations in VGLUT-1 and -2 expression almost completely recovered at 1 month after earplug removal. Our results suggested that the cell size and VGLUT expression in the CN have a neuroplasticity capacity, which is regulated by increases and decreases in sound levels. Restoration of the sound levels might partly prevent cell size decrease and maintain VGLUT expression in the CN.

## 1. Introduction

Sound stimuli are relayed to the auditory center from the cochlea to the cochlear nucleus (CN). Transmitted sound waves reaching the cochlea are converted to an equivalent electrical signal in the cochlear hair cells (HCs). Spiral ganglion neurons (SGNs) are the primary auditory neurons, and they contribute to signal transmission from the HCs to the CN. There are two types of SGNs, type I and type II. Type I SGNs represent approximately 95% of SGNs and form synapses with inner HCs, whereas type II SGNs synapse with outer HCs [1]. The main excitatory activity in type I SGN pathways is glutamatergic [2]. The CN is the first auditory relay point where auditory inputs are integrated with other sensory inputs, such as somatosensory and vestibular input [3,4]. Vesicular glutamate transporter-1 (VGLUT-1) and transporter-2 (VGLUT-2) are expressed at the CN terminals and are reliable markers of glutamatergic synapses and neurons [5,6]. The terminals of type I auditory nerve fibers in the CN express VGLUT-1, but not VGLUT-2, whereas projections originating from other systems express VGLUT-2, but not VGLUT-1. VGLUT-1 is expressed mainly in regions of the ventral CN (VCN) that receive auditory inputs [6,7]. In contrast, VGLUT-2 is expressed mainly in regions of the granule cell lamina (GCL), which encapsulates the VCN on the dorsal and lateral sides and primarily contains granule and small cells that receive somatosensory inputs and type II SGN fibers [3,6]. 

It has been reported that cochlear insults, including acoustic overstimulation and ototoxic drugs, result in a loss of HCs and SGNs, a significant increase in VGLUT-2 expression in the GCL regions receiving non-auditory inputs, and a reduction in VGLUT-1 expression in VCN regions receiving auditory nerve inputs [3,5]. This cross-modal plasticity of VGLUT expression in the CN after cochlear insult is considered of potential clinical importance for tinnitus [8]. We previously reported on the central neural changes in the CN following cochlear lesions caused by selective HC loss without SGN damage [9]. This result might indicate that decreases in the sound levels, without primary neural damage, could cause neural changes [10]. Moreover, there are numerous studies that reported activity-dependent changes in the central auditory system at different levels (synaptic, cellular, and system levels) and across different disciplines [11,12,13]. However, the changes in VGLUT expression after decreasing the sound levels are less well known, and studying this alteration could provide valuable insights into the magnitude and spatial distribution of auditory and non-auditory innervation of the CN.

Although animal models of hearing loss are conventionally generated by noise overexposure or ototoxic drug administration, such as sensorineural hearing loss, increasing or decreasing the sound levels is difficult due to the loss of HC and SGNs, which have no regenerative capacity. Conversely, conductive hearing loss (CHL) is defined as decreased sound transmission efficiency due to external or middle ear disease, without damage to the neural component [14]. Generally, sound intensity decreases with CHL. Moreover, an animal model of CHL could be generated for controlling auditory activities by occlusion and removal of earplugs. Interestingly, a decrease in VGLUT-1 expression in CHL has previously been observed in animal models [15]. However, changes in VGLUT-2 expression levels in response to transient CHL have not been evaluated to date. Furthermore, it remains unknown whether the expression of VGLUT-1 and VGLUT-2 is fully restored following the complete resolution of CHL. In this study, we established a CHL mouse model using a custom earplug inserted into the auditory canal and examined whether decreases or increases in sound levels can induce cellular and VGLUT expression changes. We also evaluated neuroplastic adaptations in the CN, with a focus on VGLUT expression.

## 2. Experimental Procedures

### 2.1. Animals, Groups, and Ear Plugging

**Figure 1:**
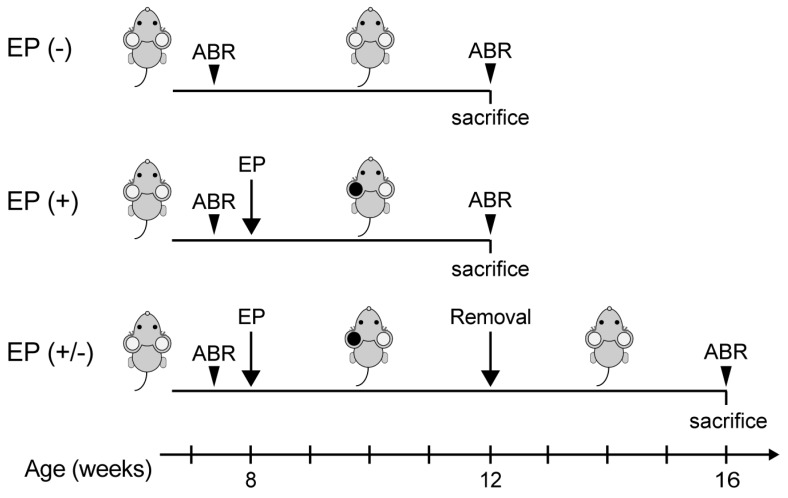
Experimental schedule. ABR, auditory brainstem response; EP, earplug.

We performed all animal experiments in accordance with the guidelines of the Animal Experimentation and Ethics Committee of the Kitasato University School of Medicine. In this study, we used 8-week-old male C57BL/6 mice with custom earplugs used to establish CHL. To place the earplug, mice were sedated using intraperitoneally injected medetomidine (0.75 mg/kg), midazolam (4 mg/kg), and butorphanol (5 mg/kg). Earplugs fabricated from a silicone compound (Otoform Ak; Dreve Brand, Hamburg, Germany) were placed in the left ear under stereomicroscopy (Leica S9E; Leica Microsystems, Tokyo, Japan). Animals were inspected every few days to ensure that the earplug remained tightly packed within the left ear canal. The earplug was re-inserted, as needed, when it was misplaced or removed. All mice needed earplug reinsertion at least once every 2 weeks. Animals were divided into three groups for analysis, as follows (Figure 1): mice without an earplug inserted, and sacrificed at 12 weeks of age (EP(-) group, *n* = 5 animals); mice with a unilateral earplug inserted into the left ear for 4 weeks, sacrificed at 12 weeks of age (EP(+) group, *n* = 5 animals), or allowed to survive for another 4 weeks after removal of the earplug and sacrificed at 16 weeks of age (EP(+/-) group, *n* = 5 animals). After sacrifice, cochleae were examined to quantify the survival of SGNs, and the transverse sections of the brainstem, through the CN, were examined for VGLUT-1 and VGLUT-2 expression and Nissl staining.

### 2.2. Auditory Brainstem Response

Auditory brainstem responses (ABRs) were used to measure the hearing thresholds of mice, as previously described [10]. Briefly, mice were anesthetized using an intraperitoneal injection of midazolam (4 mg/kg), medetomidine (0.75 mg/kg), and butorphanol (5 mg /kg). A total of 256 responses were averaged using a Neuropack Sigma system (Nihon Koden, Tokyo, Japan). ABR waveforms were recorded using tone burst stimuli at frequencies of 4, 8, 16, and 32 kHz, at 5-dB sound pressure level intervals, until no waveform could be visualized. ABRs were measured at least 1 day prior to earplug placement (baseline ABRs) and at 12 or 16 weeks, depending on the group (*n* = 5 animals per group; Figure 1). Regarding the EP(+) mice, ABR measurement performed at 4 weeks after earplug insertion indicated moderate to severe hearing loss. According to these results, the EP(+/-) mice were also assumed to have similar hearing thresholds during earplug insertion.

### 2.3. Cochlear Immunohistochemistry and Assessment

Cochlear immunohistochemistry was performed as previously described [10]. Briefly, mice were anesthetized using an intraperitoneal injection of midazolam (4 mg/kg), medetomidine (0.75 mg/kg), and butorphanol (5 mg/kg). After intracardial perfusion with 4% paraformaldehyde (PFA) in phosphate buffer (PB), cochleae were removed and fixed in 4% PFA in PB for 1 h and decalcified in 5% ethylenediaminetetraacetic acid for 1 week. Subsequently, cochleae were embedded in Tissue-Tek O.C.T (Sakura, Tokyo, Japan) and sectioned into 20-μm-thick cryostat sections. Immunohistochemistry was performed overnight at 4 °C using anti-TuJ1 (#801201; 1:200; Biolegend, San Diego, CA, USA) and anti-peripherin (#AB1530; 1:200; Millipore, Burlington, MA, USA) as primary antibodies. The sections were washed three times using PB saline (PBS) and incubated with the corresponding secondary antibody (Alexa Fluor, IgG; Invitrogen, Waltham, MA, USA) diluted 1:200 in antibody diluent. Then, the sections were mounted using an antifade mounting medium (Vectashield, Vector Laboratories, Burlingame, CA, USA) and observed under confocal laser microscopy (LSM710; Zeiss, Jena, Germany). Regarding SGN density measurements, we counted TuJ1- and peripherin-positive SGNs in the middle turn of Rosenthal’s canal in three sections per animal (*n* = 5 animals per group). ImageJ software was used to measure the area of Rosenthal’s canal, and the SGNs density per 10,000 μm^2^ was analyzed for each profile. Regarding the size assessment of the SGN cells, we measured the soma areas of TuJ1-positive SGNs in the middle turn using ImageJ in the same sections used for SGN counting. Twenty TuJ1-positive SGN cells were randomly selected in each section to measure and calculate the average cell size.

### 2.4. Brain Tissue Preparation

Mice were anesthetized as previously described. After intracardial perfusion with 4% PFA in PB, the brainstems were removed. Following this, tissues were embedded in Tissue-Tek O.C.T. and frozen transverse sections (20 µm) were prepared on glass slides. For Nissl staining, cresyl violet was used to perform stereological analysis of the CN. For each animal, three pictures were taken at equal intervals, from caudal to rostral (one picture from the 25th, one from the 50th, and one from the 75th percentile). The density and size of VCN neurons were measured using ImageJ. The number of VCN neurons per 10,000 μm^2^ was quantified (*n* = 5 animals) for the assessment of neuronal density. The average cell size in the VCN was measured in the same sections used to count cell numbers. For this purpose, 20 neurons were randomly selected in each section (*n* = 5 animals). In this analysis, we did not distinguish among VCN neuronal types.

### 2.5. Immunocytochemistry of the CN

Immunocytochemistry and quantification of the CN were conducted as previously described [9,16]. Sections were incubated for 30 min in 1% normal goat serum in PBS containing 0.3% Triton X-100 for blocking, followed by incubation with primary antibodies, anti-VGLUT1 (rabbit anti-VGLUT-1, 1:2000; Synaptic Systems, Göttingen, Germany) and anti-VGLUT-2 (rabbit anti-VGLUT-2, 1:2000; Synaptic Systems), overnight at room temperature. After washing in PBS, the sections were incubated with the secondary antibody, goat anti-rabbit Alexa Fluor (Molecular Probes, Eugene, OR, USA), diluted 1:500 in blocking buffer for 1 h. After washing in PBS, the slides were cover-slipped using an antifade mounting medium (Vectashield; Vector Laboratories). VGLUT-1 puncta density in the VCN and VGLUT-2 puncta density in the GCL were quantified (*n* = 5 animals). For each animal, three pictures were taken at equal intervals, from caudal to rostral (one picture from the 25th, one from the 50th, and one from the 75th percentile). Then, the photomicrographs were analyzed using ImageJ for automatic quantification. To ensure the reliable counting of puncta, visual inspections and manual corrections were always conducted after each automated threshold counting. This ensured that puncta were not merged by the thresholding procedure. The means and standard errors were calculated for the puncta density of VGLUT-1 and VGLUT-2 (*n* = 5 animals per group). To reduce the potential counting bias, these counting procedures were blinded to whether the tissue was obtained from normal mice or those with earplugs.

### 2.6. Statistical Analyses

Statistical analyses were performed using Graphpad Prism 8.2.1 (Graphpad Software Inc., La Jolla, CA, USA). Normality of data was verified using the Shapiro–Wilk test. For normally distributed data, we used a one-way analysis of variance (ANOVA) with correction for multiple comparisons to evaluate between-group differences using the Tukey’s post-hoc test. For non-normally distributed data, we used the non-parametric Kruskal–Wallis test, followed by Dunn’s multiple comparison test. ABR results were evaluated using two-way ANOVA, followed by Tukey’s multiple comparisons test. All data values were presented as means ± standard error. A *p* value < 0.05 was considered statistically significant. 

## 3. Results

### 3.1. ABR Assessment

**Figure 2:**
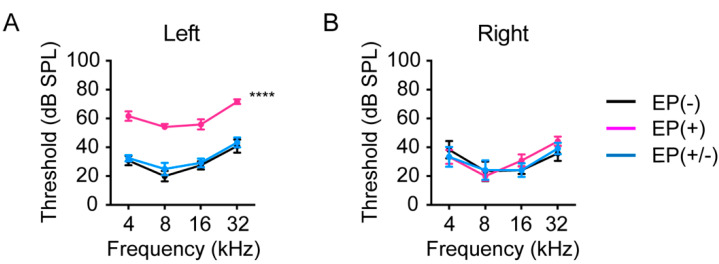
Effects of earplugs on hearing thresholds. (**A**) ABR thresholds in the EP(+) mice were significantly different from those in the EP(-) and EP(+/-) mice in the occluded left ear. ABR thresholds were measured at 12 weeks of age in the EP(-) and EP(+) mice, and at 4 weeks after EP removal in the EP(+/-) mice (n = 5 per group). (**B**) No significant differences were observed in the ABR thresholds of the right ear among the groups. ABR measurements were examined at 12 weeks of age in the EP(-) and EP(+) groups and at 16 weeks of age in the EP(+/-) group (*n* = 5 per group). EP, earplug; ABR, auditory brainstem response; SPL, sound pressure level. ****p < 0.0001.

The baseline ABR thresholds were measured in all mice (*n* = 5 per group) at 8 weeks of age before earplug placement. At baseline, no significant differences in ABR thresholds were observed among groups. Earplugs were inserted in the left ear at 8 weeks of age in the EP(+) and EP(+/-) groups. To investigate the effect of earplug insertion and removal on hearing thresholds, ABR responses were measured. In the occluded left ears, the increase in ABR thresholds, from baseline, was significantly greater in the EP(+) than in the EP(-) and EP(+/-) mice (two-way ANOVA, EP(-) versus EP(+), *p* < 0.0001; EP(+/-) versus EP(+), *p* < 0.0001; Figure 2A). However, no significant differences were observed in the ABR thresholds between the EP(-) and the EP(+/-) mice (two-way ANOVA followed by Tukey’s comparisons post hoc test, *p* = 0.47), indicating that the 1-month unilateral auditory deprivation did not induce persistent elevations in hearing threshold, in line with a previous report [17]. In the non-occluded right ears, no significant differences were observed among the groups [two-way ANOVA, *F*_(6,60)_ = 0.54, *p* = 0.78; Figure 2B].

### 3.2. Survival and Cell Size of SGNs

**Figure 3:**
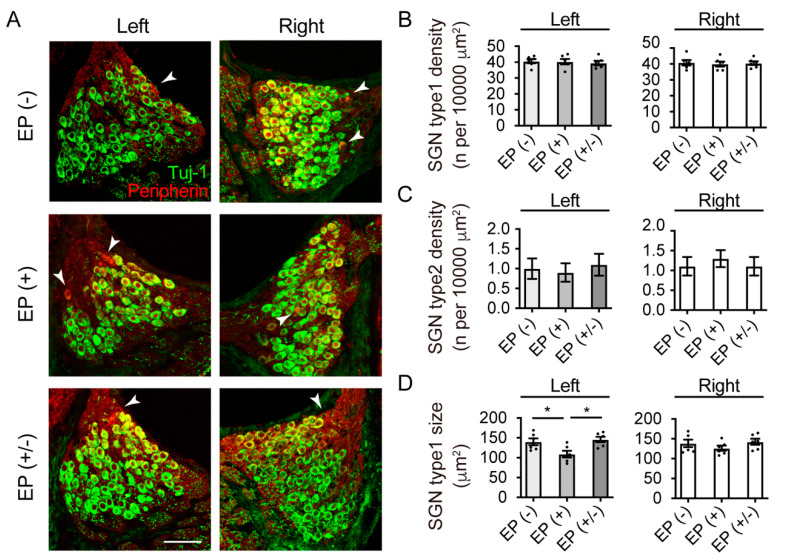
Immunofluorescence images of TuJ1 (green) and peripherin (red) in the Rosenthal’s canal and quantitative analyses. (**A**) The Rosenthal canal in all groups appeared densely packed with SGNs with a normal shape appearance. White arrows indicate the peripherin-positive SGNs. The EP(+) and EP(+/-) mice showed roughly similar SGN densities compared to those in the EP(-) group. (**B**) Type I SGN (TuJ1 positive) density indicated no statistically significant difference among groups in both ears (*n* = 5 per group). (**C**) Type II SGN (peripherin positive) density indicated no statistically significant difference among groups in both ears (*n* = 5 per group). (**D**) Quantitative analysis showed significantly smaller SGN sizes in the EP(+) than in the EP(−) and EP(+/−) mice in the left ear. No statistically significant differences were observed among the groups in the right ear. Scale bar indicates 50 μm. EP, earplug; SGN, spiral ganglion neuron. *p < 0.05.

Myelinated type I and unmyelinated type II SGNs project to the CN and form glutamatergic synapses with CN principal neurons. Type I SGN terminals express VGLUT-1 but not VGLUT-2 [6]. Before we analyzed the expression of VGLUT-1 and -2 in the CN, we investigated the effects of transient CHL on the size and number of SGNs (*n* = 5 animals per group). The frozen cochlear cross-sections were immunostained with antibodies against TuJ1 and peripherin, which are specific type I and type II SGN markers, respectively (Figure 3A). In all groups, Rosenthal’s canal was densely packed with TuJ1-positive, type I SGNs, which had a normal shape and appearance, while only a few type II SGNs were observed. The EP(+) and EP(+/-) mice had roughly similar SGN densities as those in the EP(-) mice in both ears. The neural density of type I and type II SGNs were not significantly different across all experimental groups in both ears (type I, left ear: *F*_(2,15)_ = 0.25, *p* = 0.78; type I, right ear: *F*_(2,15)_ = 0.16, *p* = 0.85; one-way ANOVA; Figure 3B; type II, left ear: *p* = 0.85; type II, right ear: *p* = 0.77; Kruskal–Wallis test; Figure 3C). Regarding SGN cell size in the left ear, the EP(+) mice showed significantly smaller SGNs than the EP(−) (one-way ANOVA; EP(+) vs. EP(−), *p* = 0.04; Figure 3D) and EP(+/−) mice (one-way ANOVA; EP(+) vs. EP(+/−), *p* = 0.01; Figure 3D). However, the SGN sizes did not differ between the EP(−) and EP(+/−) mice (one-way ANOVA; EP(−) vs. EP(+/−), *p* = 0.87; Figure 3D). In the right ear, no significant differences were observed in SGN cell size across all experimental groups (one-way ANOVA; *F*_(2,15)_ = 0.25, *p* = 0.78; Figure 3D).

### 3.3. Cellular Assessment in the CN

**Figure 4:**
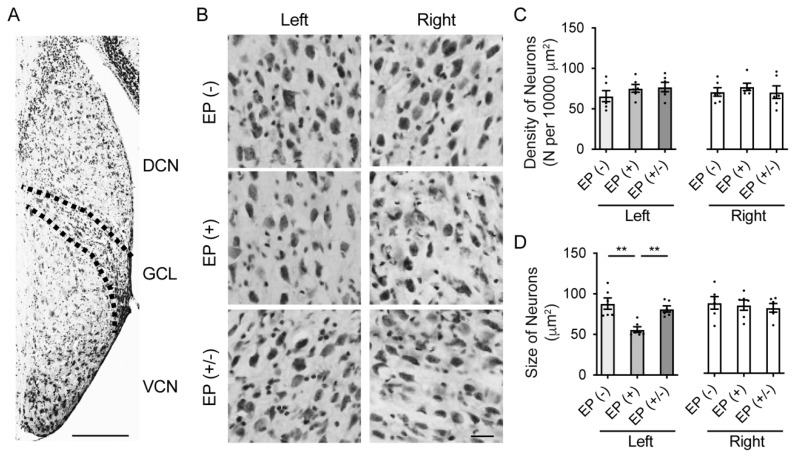
Stereological analysis in the VCN and quantitative analyses of cell density and size. (**A**) Representative images of low magnification stained with cresyl violet show the area of the DCN, GCN, and VCN in the EP(-) mice. Scale bar indicates 200 μm. (**B**) Representative images of the VCN in high magnification showed the characteristics of VCN cells in each group. Scale bar indicates 50 μm. (**C**) Quantitative analysis showed that the density of neurons was not significantly different among groups in both ears. (**D**) Quantitative analysis showed that the neuron size in the VCN for the right ear was not significantly different among groups; however, neuron size was significantly smaller in the left ear of the EP(+) mice than in the EP(-) and EP(+/-) mice. EP, earplug; DCN, dorsal cochlear nucleus; GCL, granule cell lamina; VCN, ventral cochlear nucleus. **, *p* < 0.01.

To investigate the stereological cellular changes in the VCN, including the neuronal density and cell size, after auditory deprivation post earplug insertion, transverse brainstem sections were examined for Nissl staining (*n* = 5 animals per group; Figure 4A,B). The neural density of the VCN showed no significant differences among groups in both ears (left ear: *F*_(2,15)_ = 0.51, *p* = 0.61; right ear: *F*_(2,15)_ = 0.66, *p* = 0.53; one-way ANOVA; Figure 4C). However, in the occluded left ears, the cell size was significantly smaller in the EP(+) than in the EP(-) and EP(+/-) mice (one-way ANOVA, EP(-) vs. EP(+), *p* = 0.001; EP(+/-) vs. EP(+), *p* = 0.007; Figure 4D). However, no significant difference in cell size was observed between the EP(+/-) and EP(+) mice (one-way ANOVA, *p* = 0.63). In the non-occluded right ears, there was no significant difference in cell size across all experimental groups (one-way ANOVA, *F*_(2,15)_ = 0.21, *p* = 0.81).

### 3.4. VGLUT Expression in the CN

**Figure 5:**
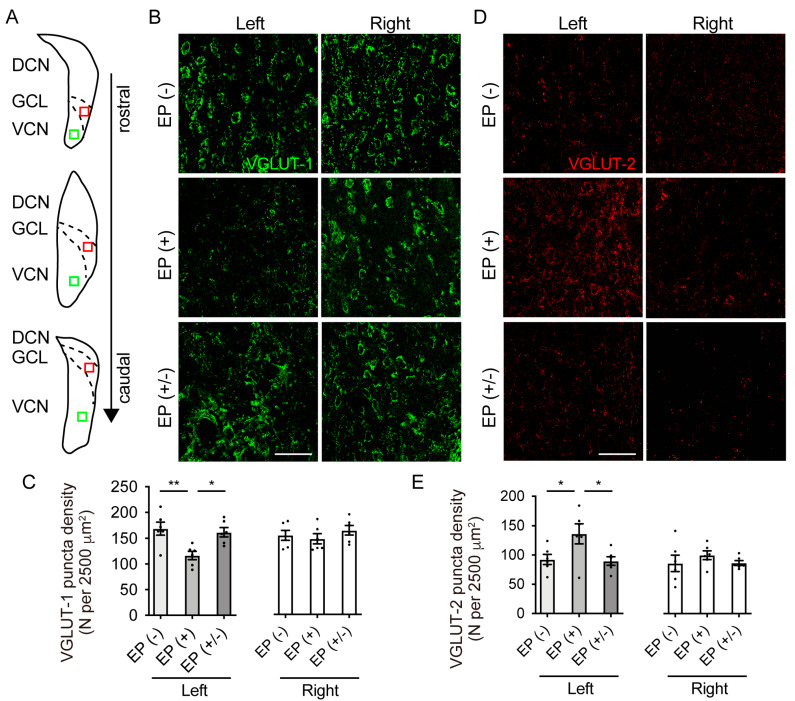
VGLUT-1 and VGLUT-2 expression in the VCN and quantitative analyses of puncta density. (**A**) Schematic view of the right cochlear nucleus (CN) regions indicates the locations where photomicrographs were taken for the analysis of VGLUT-1 (green box) and VGLUT-2 (red box). (**B**) Representative images of the VCN stained with VGLUT-1 show that puncta density in the right ear was similar in all mice, but lower in the left ear of the EP(+) mice. Scale bar indicates 50 μm. (**C**) VGLUT-1 puncta density in the right ear showed no significant difference among groups, but was significantly lower in the left ear of the EP(+) than in the corresponding ear of the EP(-) and EP(+/-) mice (*n* = 5 animals in each group). (**D**) Representative images of the GCL stained with VGLUT-2 show that the puncta density of the right ear in all mice was similar, but higher in the left ear of the EP(+) mice. Scale bar indicates 50 μm. (**E**) VGLUT-2 puncta density in the right ear showed no significant difference among groups but was significantly higher in the left ear of the EP(+) mice than in the EP(-) and EP(+/-) mice (*n* = 5 animals per group). DCN, dorsal cochlear nucleus; GCL, granule cell lamina; VCN, ventral cochlear nucleus; VGLUT-1, vesicular glutamate transporter-1; VGLUT-2, vesicular glutamate transporter-2. **, *p* < 0.01; *, *p* < 0.05.

To determine whether CHL alters the expression of glutamatergic synaptic markers in the CN, the CN was immunostained using antibodies against VGLUT-1 and VGLUT-2 (*n* = 5 animals per group; Figure 5A,C). VGLUT-1 puncta density in the VCN was statistically significantly different among groups in the occluded left ears, but not in the non-occluded right ears (left ear: *F*_(2,15)_ = 7.88, *p* = 0.005; right ear: *F*_(2,15)_ = 0.75, *p* = 0.49; one-way ANOVA; Figure 5B). In the occluded left ears, VGLUT-1 puncta density was significantly lower in the EP(+) mice than in the EP(-) and EP(+/-) mice (one-way ANOVA, EP(-) vs. EP(+), *p* = 0.006; EP(+/-) vs. EP(+), *p* = 0.017). However, no significant difference was observed between the EP(+/-) and EP(+) mice (one-way ANOVA, *p* = 0.87). Furthermore, quantitative analysis of VGLUT-2 puncta density in the GCL revealed significant differences among the groups in the occluded left ears, but not in the non-occluded right ears (left ear: *F*_(2,15)_ = 4.9, *p* = 0.02; right ear: *F*_(2,15)_ = 0.68, *p* = 0.52; one-way ANOVA; Figure 5D). In the occluded left ears, VGLUT-2 puncta density was significantly higher in the EP(+) than in the EP(-) and EP(+/-) mice (one-way ANOVA, EP(+) vs. EP(-), *p* = 0.048; EP(+) vs. EP(+/-), *p* = 0.036). However, no significant difference was observed between the EP(+/-) and EP(+) mice (one-way ANOVA, *p* = 0.99).

## 4. Discussion

This study revealed that a decrease in sound levels by CHL leads to significant reduction in neuronal cell size and cross-modal synaptic alteration in VGLUT expression in the CN, despite the lack of neuronal loss in the SGNs and the CN. Surprisingly, almost all cellular and VGLUT expression changes fully recovered at 1 month post earplug removal in the EP(+/-) mice. Importantly, our findings suggested that the size of SGNs and VCN neurons and VGLUT expression in the CN exhibit a neuroplasticity capacity, which is regulated by increases and decreases in sound levels. Restoration of sound levels might partly prevent these cell size decreases and maintain VGLUT expression in the CN.

CHL did not lead to a decrease in the number of SGNs and CN neurons, indicating that no neuronal death was caused by the decrease in sound levels by ear plugging over the time course of this study. After development of peripheral cochlear lesions, such as those caused by noise overexposure, neural loss might progress very slowly over years. Therefore, considering the relatively short duration of our study, we cannot exclude the possibility of long-term neuronal death, including death of SGNs and VCN neurons after CHL. Future studies with long-term observation after CHL are needed to elucidate whether SGNs and VCN neurons could survive throughout life with CHL. Furthermore, the mice with an inserted earplug exhibited significantly smaller SGN and VCN neuron sizes than those without earplugs, and the cell size almost fully recovered at 1 month after earplug removal, indicating that it might be regulated by the hearing levels. Our results were consistent with previous reports indicating that the size of SGNs and VCN neurons decreases after blockade of SGN electrical activity [18,19]. Therefore, the reduction in SGN and VCN neuron size after cochlear insults is not only due to direct auditory neural damage but also to decreased sound levels. Moreover, a previous study reported that this reduction is followed by delayed neural death [20]. The decrease in the sizes of SGNs and VCN neurons observed in our study, despite their survival, suggested that the relationship between cell size and survival in these neurons is complex and influenced by multiple factors. Further experiments will be necessary to elucidate the mechanisms underlying the decrease in size of SGNs and VCN neurons and the relationship between decreased VGLUT-1 expression and decreased neural size or sound levels.

Although mice with earplugs showed no significant loss of SGNs and VCN neurons, VGLUT-1 expression decreased significantly in the VCN, which receives type I SGN projections, consistent with previous reports of cochlear damage by noise overexposure or ototoxic drug injection [3,5]. Moreover, CHL reduces the number of VGLUT-1 and glutamate molecules per synaptic vesicle, which is likely to lead to decreased vesicular glutamate release [15,21]. A recent study reported that decreased VGLUT-1 puncta density in the CN region is not necessarily accompanied by SGN degeneration, consistent with our results showing decreased VGLUT-1 expression without SGN degeneration [16]. Other studies have suggested that VGLUT-2, a marker for non-auditory neurons, is up-regulated in the GCL regions to compensate for cochlear damage, indicating a re-innervation of the CN by inputs from the non-auditory system in response to decreased auditory inputs [3,5,16,22]. VGLUT-1 and VGLUT-2 expression showed cross-modal plasticity in the CN, which might be important for tinnitus [23,24]. We observed cross-modal alterations of VGLUT expression, a synaptic marker expressed in the EP(+) mice, indicated by decreased VGLUT-1 expression in the VCN and increased VGLUT-2 expression in the GCL. These results suggested that the increased non-auditory input to the GCL, after decreasing the sound levels, might result in tinnitus. Thus, the maintenance of hearing levels is important to control synaptic plasticity and tinnitus.

Ipsilateral CHL could affect molecular changes in the contralateral CN, with these contralateral neurons exhibiting increased metabolic rates and protein synthesis [25,26]. However, we did not observe a change in the contralateral VGLUT expression in our ipsilateral model of CHL. It is unclear whether the difference in these results is caused by the duration of CHL, use of a different strain or age of mice, and/or the timing of analysis after CHL. Further detailed studies are needed to determine how the contralateral CN changes in response to ipsilateral CHL.

Globally, there are numerous patients with various types of CHL, including cholesteatoma and chronic otitis media. Several treatment options, including medication, surgery, and hearing aids, have been considered to restore the auditory function in patients with CHL. This study provides evidence that untreated CHL leads to cell size decrease and alterations of VGLUT expression in the CN. Our results indicated that hearing improvement by surgical treatment or hearing aids would be reasonable for full restoration of hearing thresholds and cellular and VGLUT expression recovery in the CN. 

Our auditory deprivation model also allowed a more in-depth investigation of the mechanism of central neural plasticity and the regulation of VGLUT expression in the auditory pathway. Likewise, our study carries significant clinical implications for the treatment of patients with CHL. However, our study had limitations. The observation time course of this study was relatively short. Therefore, we could not determine whether central auditory neuroplasticity in the CN occurred similarly after long-term CHL. Future studies with long-term follow-up of CHL are required to elucidate the auditory activity dependent on the neural change and capacity of auditory neuroplasticity.

## 5. Conclusions

We found that the decreased sound levels due to auditory deprivation significantly decreased the cell size and VGLUT-1 expression in the VCN, and increased VGLUT-2 expression in the GCL, despite the survival of the SG and VCN neurons. At 1 month after restoration of the sound levels by resolution of deprivation, almost all anatomical and VGLUT expression alterations were fully reversed, suggesting that auditory activities are important to maintain central auditory capacity, which shows that auditory activity is dependent on plasticity in the CN. Our findings are expected to contribute to the mechanisms of auditory-dependent plasticity clarification. Therefore, restoration of the sound levels in patients with hearing loss might partly contribute to the prevention of neural changes in the CN and preservation of auditory functions.
